# Puerperal Eclampsia, Forceps Delivery and Venesection

**Published:** 1882-04

**Authors:** 


### ARTICLE VIII.

LINDEN, WIS., March 22, 1882.

### PUERPERAL ECLAMPSIA FORCEPS DELIVERY AND VENESECTION.

Mrs. S., a young English woman, aged 18, and a very plethoric primipara, was seized without any previous symptoms, at seven P. M., January 16, 1882, with a severe convulsion, which, according to the opinion of her medical attendant, lasted some minutes. I was sent for immediately, and arrived at the house in about twenty minutes after the first spasm. Upon my arrival I found her in one of the most severe convulsions possible. Her respiration had so far ceased that her face was nearly black. Her tongue was badly lacerated, and protruding through oozing froth and blood. Radial pulse scarcely perceptible.

I caused her to be placed on a bed at once and opened the median basilic vein, taking about six ounces of very dark blood. As soon as this amount of blood had been taken the convulsions began to diminish, although the respiration was slow and labored. I then administered chloroform, after which I made examination per vaginam and found the os fully dilated with the head engaged and child living. I think I never saw a case where the fundus of the uterus was so hard and contractions so constant. I placed the patient upon the edge of the bed with knees flexed, and applied my Miller forceps at once. After which forceps being tied and locked, patient was placed in her old position and covered. Finding some little cessation of the uterine contractions about this time, I waited until the next contraction, when I made traction, and in less than twenty minutes from the time I entered the house I delivered her of a living girl baby weighing eleven pounds. Shortly after the placenta presented and was expelled almost entirely by uterine contractions, but immediately upon the delivery of placenta another severe convulsion came on. I will add here that there was very little uterine hæmorrhage. I then administered chloroform and sent for Dr. Eastman, of Mineral Point, seven miles away. Meantime I kept her under the influence of chloroform, more or less. I introduced the catheter and drew off about four ounces of urine, which I tested, and found it to contain a large per cent. of albumen. Later tests showed a specific gravity of 1007. Dr. Eastman arrived about 10 p. m. We at once discontinued the use of the chloroform. Shortly after she had another convulsion, lasting three minutes. Upon consultattion we decided to take more blood, and did so before the convulsion ceased. The spasm seemed to subside at once when blood was taken. The amount of blood taken at that time was about thirty ounces, after which I administered as follows :

℞ Chloral hydrate.................. ʒj.

Aqua.............................. ℥j.

M. and S. One teaspoonful every hour in a tablespoonful of water.

No convulsive efforts of any importance were afterward seen.

Acting upon the supposition that the eclampsia was caused by chemical changes in the blood, we took away largely of the same, and after that diluted it as freely as the stomach could take it with copious draughts of water. The patient made a rapid and good recovery, and at present is quite well and has a healthy child.

Summing up, then, I would venture the opinion that my patient was suffering from the toxic effects of whatever poison is generated by the non-action of the kidneys. The elements of success consisted, 1st, in the immediate delivery of the child, thus lessening the pressure on the kidneys. 2d. Diminishing the amount of blood, thus lessening the quantity of the poison in the system. 3d. Diluting the remaining blood with water, thus rendering it less potent in its effects on the nerve centers.

---

FEMALE DOCTORS IN RUSSIA.—The number of female candidates for entering upon the study of medicine in Russia is increasing. The normal number of admissions is fixed at seventy, but the actual number of applicants now amounts to more than double that number. The total number of female students who have been admitted within ten years was 959, and of these 281 appear to have terminated their studies and been admitted to the practice of medicine.—*Med. and Surg. Reporter.*

---

THE DANGERS OF NERVE STRETCHING.—Five fatal cases of nerve-stretching for locomotor ataxia are reported; one by Socin; one by Langenbeck (who originated the operation); one by Billroth and Weiss; one by Berger, and one by Benedict. In these cases it appeared as if there had been some violence done to the spinal cord and medulla, *e. g.*, vomiting, singultus, paralysis of bladder, etc. Dr. Althaus considers it an unsafe measure when there are cardiac or respiratory diseases.—*Med. Record.*

